# Impact of Maternal Obesity on Inhaled Corticosteroid Use in Childhood: A Registry Based Analysis of First Born Children and a Sibling Pair Analysis

**DOI:** 10.1371/journal.pone.0067368

**Published:** 2013-06-28

**Authors:** Adrian J. Lowe, Cecilia Ekeus, Lennart Bråbäck, Kristiina Rajaleid, Bertil Forsberg, Anders Hjern

**Affiliations:** 1 Occupational and Environmental Medicine, Department of Public Health and Clinical Medicine, Umeå University, Umeå, Sweden; 2 Department of Public Health and Clinical Medicine, Epidemiology and Global Health, Umeå University, Umeå, Sweden; 3 Murdoch Childrens Research Institute, Melbourne, Australia; 4 Centre for Molecular, Environmental, Genetic and Analytic Epidemiology, School of Population Health, The University of Melbourne, Melbourne, Australia; 5 Department of Woman and Child Health, Karolinska Institute, Stockholm, Sweden; 6 Centre for Health Equity Studies (CHESS), Karolinska Institutet/Stockholm University, Stockholm, Sweden; 7 Department of Research and Development, Västernorrland County Council, Sundsvall, Sweden; 8 Centre for Epidemiology, National Board of Health and Welfare, Stockholm, Sweden; 9 Clinical Epidemiology, Department of Medicine, Karolinska Institutet, Stockholm, Sweden; University of Missouri-Kansas City, United States of America

## Abstract

**Background:**

It has been proposed that maternal obesity during pregnancy may increase the risk that the child develops allergic disease and asthma, although the mechanisms underpinning this relationship are currently unclear. We sought to assess if this association may be due to confounding by genetic or environmental risk factors that are common to maternal obesity and childhood asthma, using a sibling pair analysis.

**Methods:**

The study population comprised a Swedish national cohort of term children born between 1992 and 2008 to native Swedish parents. Maternal body mass index (BMI) was measured at 8–10 weeks gestation. Unconditional logistic regression models were used to determine if maternal obesity was associated with increased risk of inhaled corticosteroid (ICS) in 431,718 first-born children, while adjusting for potential confounders. An age-matched discordant sib-pair analysis was performed, taking into account shared genetic and environmental risk factors.

**Results:**

Maternal over-weight and obesity were associated with increased risk that the child would require ICS (for BMI≥35 kg/m^2^, aOR = 1.30, 95%CI = 1.10–1.52 compared with normal weight mothers) in children aged 6–12 years. Similar effects were seen in younger children, but in children aged 13–16 years, maternal obesity (BMI≥30) was related to increased risk of ICS use in girls (aOR = 1.28, 95%CI = 1.07–1.53) but not boys (OR = 1.05, 95%CI = 0.87–1.26). The sib-pair analysis, which included 2,034 sib-pairs older than six years who were discordant for both ICS use and maternal BMI category, failed to find any evidence that increasing maternal weight was related to increased risk of ICS use.

**Conclusion:**

Maternal obesity is associated with increased risk of childhood ICS use up to approximately 12 years of age, but only in girls after this age. These effects could not be confirmed in a sib pair analysis, suggesting either limited statistical power, or the effects of maternal BMI may be due to shared genetic or environmental risk factors.

## Introduction

Obesity is related to a range of adverse health outcomes, and there is growing evidence that obesity may be linked to increased risk of asthma [1]. Maternal obesity during pregnancy has been associated with an increased risk of childhood wheeze, asthma and asthma medication use in a number of studies [2], [3], [4], [5], including our own initial study [6].

**Figure 1:**
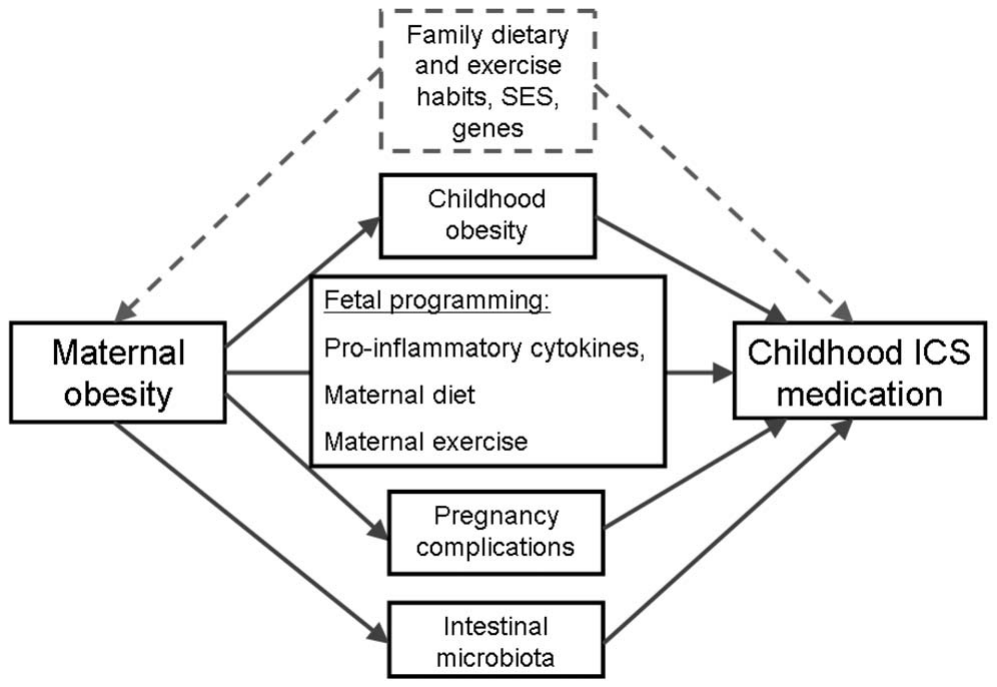
Potential explanations for the relationship between maternal BMI and increased risk of childhood asthma. Figure 1 describes the possible mechanisms, both direct and indirect, that have been proposed to explain why maternal obesity during pregnancy may increase the child’s risk of asthma. Direct effects of maternal obesity during pregnancy include altering the immune profile of the foetus by exposure to high concentrations of pro-inflammatory cytokines, or alteration of sympathetic nervous system and metabolism of brown fat, during the intra-uterine period. Indirect effects include increasing the child’s own risk of obesity, or metabolic syndrome, which may in turn increase risk of childhood asthma. The effect of maternal obesity during pregnancy may be due to increasing the risk of pregnancy complications, including pre-term birth and requirement for delivery by caesarean section. Finally, it is possible that association between maternal BMI and increased risk of asthma in the child is due to confounding by unmeasured factors, including environmental exposures (including diet, mental stress, lack of exercise, and limited sunlight exposure) and/or genetic predisposition to develop both asthma and obesity.

A range of possible mechanisms, both direct and indirect, have been proposed to explain why maternal pregnancy obesity may increase the child’s risk of asthma (figure 1). Direct effects of maternal obesity during pregnancy include altering the immune profile of the foetus by exposure to high concentrations of pro-inflammatory cytokines, or alteration of sympathetic nervous system and metabolism of brown fat [7], during the intra-uterine period. Indirect effects include increasing the child’s own risk of obesity, or metabolic syndrome [8], which may in turn increase risk of childhood asthma. The effect of maternal obesity during pregnancy may be due to increasing the risk of pregnancy complications [9], including pre-term birth [10], [11] and requirement for delivery by caesarean section [12], [13], which have been associated with increased risk of allergic disease in the child. Finally, it remains possible that the association between maternal body mass index (BMI) and increased risk of asthma in the child is due to confounding by unmeasured factors, including environmental exposures (including diet, mental stress, lack of exercise, and limited sunlight exposure) and/or genetic predisposition to develop both asthma and obesity.

In this study, we have examined the relationship between maternal BMI and risk of inhaled corticosteroid (ICS) use (as a proxy for asthma) in Swedish children. Specifically, we aimed to identify the most likely explanation for the increased risk of asthma in children of mothers with a high BMI (figure 1). Towards this end, we have conducted a within siblings-pairs analysis, to determine if change in the mothers BMI influences risk of childhood asthma. Sibling pair analysis is a powerful way of assessing unmeasured confounding, as many familial factors (both environmental exposures and genetic inheritance) are similar between siblings, but maternal BMI can change dramatically between pregnancies.

## Methods

This study is based entirely on secondary analysis of anonymised data held by the Swedish National Board of Health and Welfare and Statistics Sweden. These administrative data are collected without informed consent according to a special register legislation. Cross-linkage between registries was performed using each individual’s unique personal identification number. Swedish legislation defines the circumstances under which these data can be used for research, which includes where permission from a regional ethical committee has been granted. Permission to undertake this study was granted by the regional ethical committee of Stockholm.

### Study Population

All full term (gestational week 37-<42), singleton children born in Sweden between 1992 and 2008, according to the Swedish Medical Birth Register, were included. Only children who were residents in Sweden on December 2008 (according to the Register of the Total Population) were included. Children with one or more parents born outside of Sweden (according to the Swedish Multi-Generation Register), and those who were born pre term, were excluded as both of these factors have previously been associated with risk of allergic disease in Sweden [11], [14].

Children were excluded if they had a registration of hospital care after two years of age with a diagnosis of cerebral palsy, according to the Hospital Discharge Register, or at least one malformation reported at birth by the attending paediatrician. However, children with minor malformations (undescended testicles, preauricular appendage, congenital nevus and hip dislocation) were included. We also excluded children with a registered birth weight for gestational age greater than three or less than minus six standard deviations [15], to avoid possible coding errors.

### Measurement of Primary Exposure

Maternal BMI, calculated from measured height and weight, was obtained from the initial antenatal care visit, typically at 8–10 weeks post conception, and recorded in the Swedish Medical Birth Registry. BMI was categorised according to World Health Organization standards into underweight (<18.5 kg/m^2^), normal (18.5–24.9), overweight (25–29.9) class-1 obesity (30.0–34.9) and class-2+ obesity (*≥*35) (http://apps.who.int/bmi/index.jsp?introPage=intro_3.html).

The outcome ICS use” was defined as the purchase of at least one prescription of inhaled steroids, either by itself (Anatomical Therapeutic Chemical code starting with R03BA) or in combination with other agents (R03AK06 or R03AK07). This data was obtained from the Swedish Prescribed Drug Register, which contains all drugs prescribed and dispensed to the whole population of Sweden. There were considerable regional differences in the purchase of asthma medications, reflecting regional differences in prevalence of asthma as well as differences in access to and prescription patterns in care. A four-category *county* variable with different levels of retrieval of prescribed ICS was created to adjust the analysis for these regional differences. Dichotomised variables based on having at least one dispensed prescription of ICS during a calendar year were created for each year from 2006 to 2009. In the cohort of firstborns ICS during 2009 was used as our outcome variable, while ICS use from all four years were used to create age-equalised comparisons in the sib-pair analysis. Parental ICS was defined as receipt of an ICS during 2009 for all analyses.

### Measurement of Potential Confounders and Mediators

Information about parity, maternal age (at childbirth), mode of delivery, smoking habits in early pregnancy, maternal diseases and pregnancy complications was collected from Medical Birth Register. From the same register, perinatal data concerning infant sex, gestational age, birth weight, and low Apgar scores (<7 at five minutes) were collected. Birth weight and gestational age were used to create the indicator of small and large-for-gestational-age (<2 SD) and large-for-gestational-age (>2 SD) [15]. Maternal diseases and pregnancy complications were coded according to the International Classification of Diseases (ICD), ninth (1992–1996) and tenth (1997 and onwards) revisions.

Data on parental education was defined as the highest formal education attained by each individual as recorded in the Swedish National Education Register. Educational level was categorised by years of education into <10 years, 10–12 years, 13–14 years and >14 years. Information concerning receipt of social welfare benefits was collected from the Income and Enumeration survey for 2008 and dichotomised into any versus none.

### Statistical Methods

Two related forms of analysis were performed. In the first, all first born children were included (n = 431,718), and unconditional logistic regression models were used to assess the relationship between maternal BMI and risk of ICS use by the child during 2009, whilst adjust for standard potential confounders (age, year of birth, gender, socio-economic markers, maternal age and smoking habits). Adjustment for potential confounders was performed in a stepwise manner, with increasing model complexity. Models 1 and 2 in the unconditional logistic regression model included standard potential confounders, while model 3 included factors related to maternal obesity that may influence the child’s risk of asthma. Factors that were plausibly a result of maternal obesity, such as gestational diabetes, were added as potential mediators in model 4. Finally post birth complications (model 5) were also added.

In the second analysis, the effect of maternal BMI was assessed within sibling pairs (n = 38,058 individuals, 19,029 pairs), using conditional logistic regression (children grouped according to sharing the same mother). Where a mother had more than two children during the age range with valid data, the pair with the largest age gap was selected within a four year range. In this analysis sib age was equalised by taking ICS dispensing at the age of the youngest sib in the analysis in 2009 from the calendar year 2006 to 2008.

For all forms of analysis, potential non-linear effects of maternal BMI were assessed using the “fracpoly” command within Stata (release 10.1, College Station, Texas). Effect modification, by gender, parity, maternal smoking, parental asthma (ICS use) was assed using Wald tests. As the nature of wheeze changes with age, the exposure to asthma medications was classified into discrete age groups, and the main outcome was asthma medication use from 6–12 years, and from 13–16 years, while we report data for younger age groups in online tables for the sake of completeness.

## Results

The majority of first-born children in this cohort were born to mothers aged 25–34 years of age, and were delivered vaginally (table 1). Prescription of ICS was received by 3.6% of mothers and 3.9% of fathers during 2009, which was associated with increased risk of ICS use in the child.

**Table 1:** Prevalence of ICS use in first borns by selected socio-demographic factors, delivery details, and markers of parental asthma.

		N (431,718)	Boys (217,792)	Girls (213,926)
**Age of child (years)**	0–1	55 229	7.5%	4.8%
	2–5	105 757	7.7%	5.2%
	6–12	161 379	6.5%	4.0%
	13–16	109 353	5.5%	4.8%
**Maternal age (years)**	11–24	113 222	6.2%	4.7%
	25–34	283 586	6.8%	4.6%
	>34	34 910	6.9%	4.4%
**Maternal BMI category** **	Underweight	10 495	6.1%	4.0%
	Normal	259 269	6.4%	4.3%
	Overweight	77 169	7.0%	5.0%
	Obese class 1	20 719	7.8%	6.0%
	Obese class 2+	7 326	8.4%	6.6%
	Missing	56 740	6.5%	4.7%
**Mode of delivery**	Vaginal	319 147	6.4%	4.4%
	Elective CS	29 880	7.9%	5.6%
	Acute CS	27 621	7.3%	5.1%
	Vacuum Extraction	55 070	6.9%	4.9%
**Maternal ICS use**	No	416 117	6.4%	4.4%
	Yes	15 601	13.7%	10.2%
**Paternal ICS use**	No	414 942	6.5%	4.5%
	Yes	16 776	10.1%	7.3%
**Maternal education**	<10 years	27 013	6.7%	4.8%
	10–12	19 8183	6.6%	4.6%
	13–14	67 694	6.8%	4.7%
	15+	137 341	6.7%	4.5%
**Paternal education**	<10 years	43 002	6.7%	4.6%
	10–12	226 506	6.7%	4.7%
	13–14	63 981	6.7%	4.6%
	15+	91 112	6.6%	4.3%
	Missing	7 117		
**Gestational age (weeks)**	39–41	346 096	6.5%	4.5%
	37–38	85 622	7.3%	5.1%
**County prescription pattern** *	1	100 325	7.2%	5.0%
	2	79 221	7.0%	4.9%
	3	168 373	6.4%	4.4%
	4	83 799	6.1%	4.3%

* “County” grouped according to different levels of retrieval of prescribed ICS.

** Maternal BMI classified as Underweight (<18.5 kg/m^2^), normal (18.5–24.9), overweight (25–29.9), class-I obesity (30.0–34.9) and class-II+ obesity (≥35).

In first born singleton children aged 6–12 years of age, there was a clear increase in risk of ICS use with increasing level of maternal obesity/overweight, with the largest increase in risk being associated with having a very obese (>35 kg/m^2^) mother (table 2). Adjustment for various potential confounders did not reduce these associations (models 1–3) and additional adjustment for both pregnancy related complications (model 4) and post birth outcomes (model 5) did not influence these associations. Additional adjustment for parental ICS use did not materially alter these results. The unadjusted associations were similar in younger children (Table S1), however adjustment, particularly adjustment for maternal education and smoking, reduced some of these associations. In contrast, there was no clear effect of maternal BMI on ICS use in children aged 13–16 years (table 2).

**Table 2:** Associations between maternal BMI and inhaled cortisone for children aged 6–16 years.

6–12 year olds	Proportion	Unadjusted	Model 1	Model 2	Model 3	Model 4	Model 5
(n = 161,379)	**receiving ICS**	OR (95%CI)	OR (95%CI)	OR (95%CI)	OR (95%CI)	OR (95%CI)	OR (95%CI)
Underweight	4.5% (170/3,713)	0.92 (0.78–1.07)	0.92 (0.78–1.07)	0.94 (0.80–1.10)	0.94 (0.80–1.09)	0.93 (0.80–1.09)	0.93 (0.80–1.09)
Normal	5.0% (4802/96,559)	1.0	1.0	1.0	1.0	1.0	1.0
Overweight	5.7% (1692/29,847)	1.15 (1.08–1.22)	1.15 (1.09–1.22)	1.16 (1.09–1.23)	1.16 (1.09–1.23)	1.15 (1.09–1.22)	1.15 (1.09–1.22)
Obese Class-I	6.5% (507/7,801)	1.33 (1.21–1.46)	1.33 (1.21–1.47)	1.36 (1.23–1.49)	1.36 (1.23–1.49)	1.34 (1.22–1.48)	1.34 (1.22–1.47)
Obese Class-II+	6.7% (173/2,598)	1.36 (1.17–1.59)	1.36 (1.16–1.59)	1.38 (1.18–1.62)	1.38 (1.18–1.62)	1.36 (1.16–1.59)	1.36 (1.16–1.59)
Missing	5.2% (1,095/20,861)	1.06 (0.99–1.13)	1.06 (0.99–1.13)	0.99 (0.92–1.07)	0.99 (0.92–1.07)	0.99 (0.92–1.06)	0.99 (0.92–1.06)
**6–12 year olds**	**Proportion**	**Unadjusted**	**Model 1**	**Model 2**	**Model 3**	**Model 4**	**Model 5**
(n = 109,353)	**receiving ICS**	OR (95%CI)	OR (95%CI)	OR (95%CI)	OR (95%CI)	OR (95%CI)	OR (95%CI)
Underweight	4.9% (163/3,303)	0.94 (0.80–1.11)	0.94 (0.80–1.11)	0.95 (0.81–1.12)	0.95 (0.81–1.12)	0.95 (0.81–1.12)	0.95 (0.81–1.12)
Normal	5.2% (3,527/67,516)	1.0	1.0	1.0	1.0	1.0	1.0
Overweight	5.0% (801/15,950)	0.96 (0.89–1.04)	0.96 (0.88–1.03)	0.97 (0.90–1.05)	0.97 (0.90–1.05)	0.97 (0.90–1.05)	0.97 (0.90–1.05)
Obese Class-I	5.8% (210/3,630)	1.11 (0.97–1.29)	1.11 (0.96–1.28)	1.13 (0.98–1.31)	1.13 (0.98–1.31)	1.13 (0.98–1.31)	1.13 (0.98–1.31)
Obese Class II+	5.8% (56/965)	1.12 (0.85–1.47)	1.11 (0.85–1.46)	1.15 (0.87–1.51)	1.15 (0.88–1.51)	1.15 (0.88–1.52)	1.15 (0.87–1.51)
Missing	4.9% (884/17,989	0.94 (0.87–1.01)	0.94 (0.87–1.01)	0.94 (0.87–1.01)	0.94 (0.87–1.01)	0.93 (0.87–1.01)	0.93 (0.86–1.01)

**Model 1. Simple adjustment:** adjusted for year of birth and sex.

**Model 2. Standard potential confounders:** paternal asthma medication, socioeconomic indicators (maternal education, social welfare) maternal age, maternal smoking, county prescription pattern added.

**Model 3. Maternal pre-pregnancy confounders related to obesity:** pre-pregnancy risk factors: maternal history of diabetes and hypertension added.

**Model 4. Pre birth potential mediators:** pregnancy related complications: premature rupture of the membranes, preeclampsia, gestational diabetes, gestational hypertension, mode of delivery, gestational age (37–38 and 39–41), small and large for gestational age, maternal fever during labour, chorioamnionitis added.

**Model 5. Pre birth potential mediators:** post-birth complications of RDS, TTN, meconium aspiration added.

There was some evidence that the effect of maternal obesity during pregnancy was greater in girls than boys after age 6 years of age. Amongst 6–12 year olds, having a mother who was obese (BMI≥30 kg/m^2^) was related to a greater increase risk of ICS use in girls (OR = 1.52, 95%CI = 1.34–1.72 for model 2) than boys (OR = 1.20, 95%CI = 1.08–1.34, p for interaction <0.01). Similarly, in 13–16 year olds, maternal obesity was related to an increased risk in girls (OR = 1.28, 95%CI = 1.07–1.53 for model 2) but not boys (OR = 1.05, 95%CI = 0.87–1.26, p for interaction = 0.07). There was no evidence of modification of these effects in younger age groups, or modification by parental ICS use.

### Sibling Pair Analysis

Mothers BMI was highly correlated between pregnancies (Pearson’s r = 0.89 for children aged 6–12 years) and there was high concordance with maternal BMI category (Table S2 and Table S3). There were 7,383 sibling pairs aged 6–12 years that were discordant for ICS use, and of these 1,628 had a mother whose BMI category changed between pregnancies (Table S2). In these children, there was no evidence that high BMI in the mother during early pregnancy was related to increased risk of ICS use when examined with sibling pairs (table 3). In children aged 13–16 years, there was a trend for increasing risk with increasing level of maternal overweight and obesity, but adjustment for parity and mothers’ age reduced these associations. However, it should be noted that relatively few children could be included in this analysis (406 sibling pairs discordant for both ICS use and maternal BMI category), resulting in relatively wide confidence intervals. In the younger age groups, the unadjusted analysis suggested that obesity in the mother may be related to increased risk of ICS use (Table S4). However, these effects in younger children were confounded by maternal parity (first born children had reduced risk of ICS use up to age 6-years when compared to their younger siblings, and on average, their mother was lighter during pregnancy.

**Table 3:** Sibling pair* results for the association between Maternal BMI and inhaled cortisone - includes all discordant sib pairs aged 6–16 years.

6–12 year old pairs (n = 7,383)	Crude	Model 1	Model 2
Underweight	1.25 (0.92–1.68)	1.29 (0.95–1.76)	1.27 (0.93–1.72)
Normal	1	1	1
Overweight	0.90 (0.79–1.02)	0.90 (0.79–1.02)	0.94 (0.82–1.07)
Obese Class-I	0.94 (0.75–1.19)	0.96 (0.76–1.22)	1.06 (0.83–1.35)
Obese Class-II+	0.85 (0.58–1.23)	0.91 (0.62–1.34)	1.04 (0.70–1.55)
**13–16 year old pairs (n = 2,006)**	**Crude**	**Model 1**	**Model 2**
Underweight	1.01 (0.59–1.72)	1.01 (0.59–1.73)	1.03 (0.60–1.76)
Normal	1	1	1
Overweight	1.32 (1.03–1.68)	1.28 (1.00–1.63)	1.14 (0.89–1.47)
Obese Class-I	1.50 (0.90–2.48)	1.40 (0.84–2.33)	1.10 (0.65–1.86)
Obese Class-II+	2.33 (0.88–6.17)	2.10 (0.79–5.60)	1.52 (0.56–4.12)

* Analysis based on a conditional logistic regression model.

**Model 1**– adjusted for infant gender.

**Model 2**– also adjusted for parity and maternal age (as continuous exposure).

## Discussion

This is the largest study to date of the effect of maternal BMI on risk of asthma in the child, including all full term Swedish children born over a 16-year period. In first-born children, we observed a clear increase in risk associated with higher maternal BMI, but in older children (after age 6 years), the effect was greater in girls than in boys. There was no evidence of an effect in boys after 13 years of age. Finally, when controlling for sharing the same mother, there was no apparent effect of increased maternal BMI within a sibling pair.

This study substantially builds on our previous assessment of the relationship between maternal BMI and childhood risk of asthma [6]. Specifically, we were able to include markers of socio-economic status (parental education and receipt of social welfare), and regional variations in ICS prescription patterns, to see if these may confound or explain part of the observed relationship. We were also able to collect information on parental ICS use as a marker of parental asthma. We elected not to adjust for this within our main analysis because of the known relationship between obesity and asthma symptoms in adults [1]. Additional adjustment for parental ICS use did not materially alter the observed associations. We also limited our study population to full term Swedish children to obtain a homogeneous sample, and to ensure that the effects observed were not due to maternal obesity increasing risk of pre-term birth, a known risk factor for asthma [11].

Overall, these results support a possible role for high maternal BMI on increasing risk of asthma in the child, particularly in females. The effect appears to dissipate in boys in teenage years. Adjustment for socio-economic status, pregnancy complications and post birth respiratory complications had no effect on these associations, which is consistent with previous findings [3], [4], [6], suggesting that these factors do not explain the link between high maternal BMI and risk of asthma in the child.

The use of a sib pair design in this study is powerful technique, as it controls for unmeasured potential confounders that are common between siblings, including genetic risk factors and many environmental exposures. The lack of effect of maternal obesity on childhood asthma seen in our sib pair analysis may indicate that the effect of maternal BMI is not due to a direct effect of maternal pregnancy obesity, such as fetal programing of the infants immune or metabolic system, as has been suggested by some authors [7], [8]. However, despite using data from the whole Swedish population for a 16 year period, relatively few children could be included, as the analysis requires a) a mother to contribute two or more children who b) are discordant for use of ICS at the same age and c) for the mothers weight category to have shifted between pregnancies. In total, across all age groups studied, 3,984 sibling pairs were informative to these results. This resulted in relatively wide confidence intervals for this form of analysis when compared to the cohort analysis. The lack of observed effect in the sib analysis makes it more likely that the effect of maternal obesity is due to either a shared genetic or environmental risk factor that increases risk of both maternal obesity and childhood asthma. One possible shared risk factor is a lifestyle of high caloric intake diet and low exercise, which leads to increased risk of maternal obesity, increased risk of obesity and asthma in the child. In support of this concept are observations that high fat meals, particularly meals high in trans-fats, increase neutrophilic airway inflammation and reduce broncho-dilator response in participants with asthma [16], and that obesity has been linked to increase risk of subsequent asthma in both children [17] and adults [18]. Interestingly, in those studies that have been able to adjusting for the child’s own BMI, in childhood [2] and adolescence [19], the relationship between maternal obesity and asthma risk in the child was largely unchanged. This suggests that this relationship is not simply due to obese mothers being more likely to have an obese child, and it is the obesity in the child that puts them at increased risk of asthma. An alternate explanation for these relationships is that obesity is related to altered microbial colonisation (reduced proportion of Bacteroidetes [20]), and these alterations are passed onto the infant during birth and breastfeeding, and it is the altered colonisation of the infant gut that influences the risk of asthma [21].

When interpreting these results, it is important to consider that Sweden has a relatively low rate of obesity [22]. It has been estimated that 12.4% of Swedish women were obese in 2010, compared to 26.3%, 29.1% and 48.3% of women from the United Kingdom, Australia and American respectively. The associations observed in this study may be more important for societies where more mothers are affected by obesity.

This study has a number of important strengths and limitations. The large number of children that could be included in this study, and the ability to perform a sibling pair analysis, are substantial strengths of this work. As noted, despite the number of children included, relatively few could be included in the sib pair analysis, making the results less than definitive. Also, as we only had access to four years of prescription medication data, we could not include comparisons of siblings with more than a three year age gap, whilst still matching sibs on age, and larger age gaps would allow for greater changes in maternal BMI. It is important to match sibs for age because of the rapid decline in prevalence of asthma symptoms, and therefore ICS use, with increasing age.

We have used prescription of inhaled corticosteroids (ICS) to define current symptoms of asthma, which is clearly not the same as the presence of current wheeze or diagnosed asthma. Defining asthma in epidemiological studies remains a vexed issue. A number of studies have assessed the validity of prescription registry based information on asthma medicine use against parent report of asthma [23], standardised questionnaire based definitions of asthma [24], and doctor diagnosis [25] in similar settings to the current study. These studies generally find that prescription for ICS is a reasonable marker for asthma symptoms. Regional prescription patterns could confound associations between BMI and asthma in Sweden, because of the considerable regional differences for BMI as well as access to specialist and primary care. However, we did not find evidence that these regional differences were a major source of confounding in this study. A further limitation is that we do not have any information concerning the child’s own BMI, so we cannot assess the potential mediating or confounding effect of obesity in the child on these relationships.

The observation that high maternal BMI has a greater effect on girls than boys after the age of six years is intriguing. To our knowledge, only our own previous study on this topic [6], which was based on similar data but restricted to children born in Stockholm, has addressed the potential modifying influence of gender on the association between maternal BMI and childhood asthma risk, where we noted a similar gender specific effect. There are a range of differences between boys and girls on the expression of asthma symptoms, with clear sex differences in the prevalence of symptoms changing with age being noted in numerous studies [26]. Furthermore, some [27], [28], but not all [29], studies have found that there is a closer relationship between asthma symptoms and obesity in females than males. While the reasons for the observed gender differences are unclear, hormonal factors could be important. Obesity is associated with an earlier puberty in girls [30] and early menarche has also been related to an increased risk of asthma in women [31]. Shared genes may contribute to the association between asthma and obesity [32] and this is possibly confined to females [33]. There are profound changes in body composition between genders that occur during puberty, with females gaining significantly more fat mass during this period than males [34]. This potentially makes adipose tissue, and its metabolism and related hormones, more important for girls in the expression of asthma symptoms.

### Conclusions

In conclusion, we found a clear, dose response relationship between the degree of maternal over-weight and obesity during early pregnancy and increased risk of asthma in the child, but not in boys after the age of 13 years. The lack of an effect in the sib-pair analysis, however, favors genetic and shared environment risk factors to explain this association rather than intra-uterine programming.

## Supporting Information

Table S1 **Associations between maternal BMI and inhaled corticosteroid for children aged 0–5 years.**
(DOC)

Table S2 **Association between Maternal BMI and inhaled corticosteroid at Least Once in Discordant Sib-pairs aged 0–5 years.**
(DOC)

Table S3 **Association between maternal BMI and inhaled corticosteroid at least once in discordant sib-pairs aged 6–16 years.**
(DOC)

Table S4 **Sibling pair results for the association between maternal BMI and inhaled corticosteroid - includes all discordant sib pairs aged 0–5 years.**
(DOC)
