# Supplementary material for: Impact of Maternal Obesity on Inhaled Corticosteroid Use in Childhood: A Registry Based Analysis of First Born Children and a Sibling Pair Analysis
Source: PLoS One. 2013 Jun 28;8(6):e67368. doi: 10.1371/journal.pone.0067368 (PMC3696102; doi:10.1371/journal.pone.0067368)
Supplement: Table S1 — Associations between maternal BMI and inhaled corticosteroid for children aged 0–5 years. (DOC) [file pone.0067368.s001.doc]

## Table S1: Associations between maternal BMI and inhaled corticosteroid for children aged 0-5 years

| **0-1 year olds** | **Proportion** | | **Unadjusted** | **Model 1** | **Model 2** | **Model 3** | | **Model 4** | **Model 5** |
| --- | --- | --- | --- | --- | --- | --- | --- | --- | --- |
| **(n= 55,229)** | **receiving ICS** | | **OR (95%CI)** | **OR (95%CI)** | **OR (95%CI)** | **OR (95%CI)** | | **OR (95%CI)** | **OR (95%CI)** |
| Underweight | 5.7% (71/1241) | 0.99 (0.77-1.26) | | 1.00 (0.78-1.28) | 0.91 (0.71-1.16) | 0.91 (0.71-1.17) | 0.91 (0.71-1.17) | | 0.91 (0.71-1.17) |
| Normal | 5.8% (1925/33 220) | 1.0 | | 1.0 | 1.0 | 1.0 | 1.0 | | 1.0 |
| Overweight | 6.3% (694/10 982) | 1.10 (1.00-1.20) | | 1.10 (1.01-1.20) | 1.09 (1.00-1.19) | 1.09 (0.99-1.19) | 1.07 (0.98-1.18) | | 1.07 (0.98-1.18) |
| Obese Class-I | 7.9% (259/3266) | 1.40 (1.22-1.60) | | 1.39 (1.22-1.60) | 1.33 (1.16-1.52) | 1.32 (1.15-1.51) | 1.28 (1.12-1.48) | | 1.28 (1.12-1.47) |
| Obese Class-II+ | 7.8% (107/1365) | 1.38 (1.13-1.69) | | 1.38 (1.13-1.69) | 1.28 (1.04-1.58) | 1.26 (1.02-1.55) | 1.22 (0.99-1.50) | | 1.22 (0.99-1.50) |
| Missing | 6.9 (358/5155) | 1.21 (1.08-1.36) | | 1.12 (1.00-1.26) | 1.05 (0.88-1.24) | 1.04 (0.88-1.24) | 1.04 (0.87-1.23) | | 1.04 (0.87-1.23) |
| **2-5 year olds** | **Proportion** | | **Unadjusted** | **Model 1** | **Model 2** | **Model 3** | | **Model 4** | **Model 5** |
| **(n= 105,757)** | **receiving ICS** | | **OR (95%CI)** | **OR (95%CI)** | **OR (95%CI)** | **OR (95%CI)** | | **OR (95%CI)** | **OR (95%CI)** |
| Underweight | 5.7% (127/2238) | 0.93 (0.78-1.12) | | 0.93 (0.78-1.12) | 0.93 (0.77-1.12) | 0.93 (0.77-1.12) | 0.92 (0.76-1.11) | | 0.92 (0.77-1.11) |
| Normal | 6.1% (3758/61974) | 1.0 | | 1.0 | 1.0 | 1.0 | 1.0 | | 1.0 |
| Overweight | 7.0% (1429/20390) | 1.17 (1.10-1.24) | | 1.17 (1.10-1.25) | 1.17 (1.10-1.25) | 1.17 (1.09-1.24) | 1.16 (1.08-1.24) | | 1.16 (1.09-1.24) |
| Obese Class-I | 7.5% (453/6022) | 1.26 (1.14-1.39) | | 1.26 (1.14-1.39) | 1.26 (1.13-1.39) | 1.25 (1.12-1.38) | 1.23 (1.09-1.37) | | 1.23 (1.11-1.37) |
| Obese Class-II+ | 8.9% (214/2398) | 1.50 (1.31-1.75) | | 1.52 (1.31-1.75) | 1.50 (1.30-1.73) | 1.48 (1.28-1.72) | 1.45 (1.20-1.68) | | 1.45 (1.25-1.67) |
| Missing | 6.6% (841/12735) | 1.10 (1.01-1.18) | | 1.10 (1.02-1.19) | 1.04 (0.95-1.14) | 1.04 (0.95-1.14) | 1.03 (0.94-1.13) | | 1.03 (0.94-1.13) |

**Model 1. Simple adjustment:** adjusted for year of birth and sex

**Model 2. Standard potential confounders:** paternal asthma medication, socioeconomic indicators (maternal education, social welfare) maternal age, maternal smoking, county prescription pattern added

**Model 3. Maternal pre-pregnancy confounders related to obesity:** pre-pregnancy risk factors: maternal history of diabetes and hypertension added

**Model 4. Potential mediators:** pregnancy related complications: premature rupture of the membranes, preeclampsia, gestational diabetes, gestational hypertension, mode of delivery, gestational age (37-38 and 39-41), SGA, LGA, maternal fever during labour, chorioamnionitis added

**Model 5. Post birth potential mediators:** post-birth complications of RDS, TTN, meconium aspiration added.
