# Supplementary material for: Impact of Maternal Obesity on Inhaled Corticosteroid Use in Childhood: A Registry Based Analysis of First Born Children and a Sibling Pair Analysis
Source: PLoS One. 2013 Jun 28;8(6):e67368. doi: 10.1371/journal.pone.0067368 (PMC3696102; doi:10.1371/journal.pone.0067368)
Supplement: Table S2 — Association between Maternal BMI and inhaled corticosteroid at Least Once in Discordant Sib-pairs aged 0–5 years. (DOC) [file pone.0067368.s002.doc]

## Table S2. Association between maternal BMI and inhaled corticosteroid at least once in discordant Sib-pairs aged 0-5 years.

|  | **Sib-pairs aged 0-1 years discordant on use of ICS (n=3,472)** | | | | | | |
| --- | --- | --- | --- | --- | --- | --- | --- |
|  |  | Maternal BMI for the sib who did not use ICS | | | | | |
|  |  | Underweight | Normal | Overweight | Obese Class I | Obese Class II+ | Total |
| Maternal BMI for the sib who used ICS | Underweight | 0.9% (31) | 0.5% (16) | 0.0% (0) | 0.0% (0) | 0.0% (0) | 1.4% (47) |
|  | Normal | 1.0% (33) | 53.5% (1856) | 5.2% (182) | 0.2% (6) | 0.0% (0) | 59.8% (2077) |
|  | Overweight | 0.0% (0) | 6.1% (212) | 16.2% (562) | 2.6% (89) | 0.1% (5) | 25.0% (868) |
|  | Obese Class I | 0.0% (0) | 0.1% (2) | 3.3% (116) | 5.5% (190) | 0.6% (20) | 9.4% (328) |
|  | Obese Class II+ | 0.0% (0) | 0.0% (1) | 0.1% (4) | 1.1% (37) | 3.2% (110) | 4.4% (152) |
|  | Total | 1.8% (64) | 60.1% (2087) | 24.9% (864) | 9.3% (322) | 3.9% (135) | 100% (3472) |
|  | **Sib-pairs aged 2-5 years discordant on use of ICS (n=6,167)** | | | | | | |
|  |  | Maternal BMI for the sib who did not use ICS | | | | | |
|  |  | Underweight | Normal | Overweight | Obese Class I | Obese Class II+ | Total |
| Maternal BMI for the sib who used ICS | Underweight | 1.1% (69) | 0.7% (46) | 0.0% (0) | 0.0% (0) | 0.0% (0) | 1.9% (115) |
|  | Normal | 0.8% (50) | 55.4% (3419) | 3.6% (223) | 0.1% (4) | 0.0% (1) | 59.9% (3697) |
|  | Overweight | 0.0% (2) | 8.3% (510) | 15.6% (963) | 1.2% (74) | 0.1% (4) | 25.2% (1553) |
|  | Obese Class I | 0.0% (0) | 0.1% (7) | 3.8% (235) | 4.7% (288) | 0.6% (37) | 9.2% (567) |
|  | Obese Class II+ | 0.0% (0) | 0.0% (1) | 0.1% (7) | 1.2% (76) | 2.4% (151) | 3.8% (235) |
|  | Total | 2.0% (121) | 64.6% (3983) | 23.2% (1428) | 7.2% (442) | 3.1% (193) | 100% (6167) |
