# Supplementary material for: Impact of Maternal Obesity on Inhaled Corticosteroid Use in Childhood: A Registry Based Analysis of First Born Children and a Sibling Pair Analysis
Source: PLoS One. 2013 Jun 28;8(6):e67368. doi: 10.1371/journal.pone.0067368 (PMC3696102; doi:10.1371/journal.pone.0067368)
Supplement: Table S3 — Association between maternal BMI and inhaled corticosteroid at least once in discordant sib-pairs aged 6–16 years. (DOC) [file pone.0067368.s003.doc]

## Table S3. Association between maternal BMI and inhaled corticosteroid at least once in discordant sib-pairs aged 6-16 years.

|  | **Sib-pairs aged 6-12 years discordant on use of ICS (n=7,383)** | | | | | | |
| --- | --- | --- | --- | --- | --- | --- | --- |
|  |  | Maternal BMI for the sib who did not use ICS | | | | | |
|  |  | Underweight | Normal | Overweight | Obese Class I | Obese Class II+ | Total |
| Maternal BMI for the sib who used ICS | Underweight | 1.2% (87) | 1.3% (96) | 0.0% (0) | 0.0% (0) | 0.0% (0) | 2.5% (183) |
|  | Normal | 1.0% (75) | 57.6% (4249) | 6.6% (489) | 0.1% (11) | 0.0% (1) | 65.4% (4825) |
|  | Overweight | 0.0% (2) | 5.9% (435) | 14.4% (1060) | 2.3% (172) | 0.2% (13) | 22.8% (1682) |
|  | Obese Class I | 0.0% (0) | 0.2% (14) | 2.4% (178) | 3.5% (261) | 0.9% (68) | 7.1% (521) |
|  | Obese Class II+ | 0.0% (0) | 0.0% (1) | 0.1% (10) | 0.9% (63) | 1.3% (98) | 2.3% (172) |
|  | Total | 2.2% (164) | 64.9% (4795) | 23.5% (1737) | 6.9% (507) | 2.4% (180) | 100% (7383) |
|  | **Sib-pairs aged 13-16 years discordant on use of ICS (n= 2,006)** | | | | | | |
|  |  | Maternal BMI for the sib who did not use ICS | | | | | |
|  |  | Underweight | Normal | Overweight | Obese Class I | Obese Class II+ | Total |
| Maternal BMI for the sib who used ICS | Underweight | 1.6% (32) | 1.3% (27) | 0.0% (0) | 0.0% (0) | 0.0% (0) | 2.9% (59) |
|  | Normal | 1.3% (26) | 61.2% (1227) | 5.6% (113) | 0.0% (1) | 0.0% (1) | 68.2% (1368) |
|  | Overweight | 0.0% (0) | 7.3% (147) | 12.6% (253) | 1.6% (33) | 0.0% (0) | 21.6% (433) |
|  | Obese Class I | 0.0% (1) | 0.2% (4) | 1.6% (33) | 3.4% (68) | 0.3% (7) | 5.6% (113) |
|  | Obese Class II+ | 0.0% (0) | 0.0% (0) | 0.2% (4) | 0.4% (9) | 1.0% (20) | 1.6% (33) |
|  | Total | 2.9% (59) | 70.0% (1405) | 20.1% (403) | 5.5% (111) | 1.4% (28) | 100% (2006) |
