# Supplementary material for: Impact of Maternal Obesity on Inhaled Corticosteroid Use in Childhood: A Registry Based Analysis of First Born Children and a Sibling Pair Analysis
Source: PLoS One. 2013 Jun 28;8(6):e67368. doi: 10.1371/journal.pone.0067368 (PMC3696102; doi:10.1371/journal.pone.0067368)
Supplement: Table S4 — Sibling pair results for the association between maternal BMI and inhaled corticosteroid - includes all discordant sib pairs aged 0–5 years. (DOC) [file pone.0067368.s004.doc]

## Table S4: Sibling pair* results for the association between maternal BMI and inhaled corticosteroid - includes all discordant sib pairs aged 0-5 years.

| - 1. **year old pairs (n=3472)** | **Crude** | **Model 1** | **Model 2** |
| --- | --- | --- | --- |
| Underweight | 0.48 (0.27-0.88) | 0.51 (0.16-0.95) | 0.55 (0.29-1.01) |
| Normal | 1 | 1 | 1 |
| Overweight | 1.14 (0.94-1.38) | 1.14 (0.93-1.39) | 1.00 (0.81-1.23) |
| Obese Class I | 1.36 (0.98-1.89) | 1.39 (1.00-1.95) | 1.05 (0.74-1.48) |
| Obese Class II+ | 2.23 (1.25-3.96) | 2.39 (1.33-4.31 | 1.58 (0.87-2.87) |
|  |  |  |  |
| **2-5 year old pairs (n=6,167)** | **Crude** | **Model 1** | **Model 2** |
| Underweight | 1.23 (0.83-1.83) | 1.14 (0.76-1.72) | 1.16 (0.77-1.75) |
| Normal | 1 | 1 | 1 |
| Overweight | 1.11 (0.96-1.28) | 1.14 (0.98-1.32) | 1.05 (0.90-1.22) |
| Obese Class I | 1.23 (0.95-1.59) | 1.29 (0.99-1.68) | 1.08 (0.82-1.41) |
| Obese Class II+ | 1.57 (1.03-2.40) | 1.73 (1.12-2.67) | 1.36 (0.88-2.11) |

* Analysis based on a conditional logistic regression model.

**Model 1** – adjusted for infant gender

**Model 2** – also adjusted for parity and maternal age (as continuous exposure)
